# 226. Low incidence of *Pseudomonas* species in Gram negative blood stream infections in nine hospitals across Ecuador: an unexpected find

**DOI:** 10.1093/ofid/ofad500.299

**Published:** 2023-11-27

**Authors:** Jeannete Zurita, Gabriela Sevillano, Maria Belen Solis, Ariane Paz y Miño, Beatriz Rizkallah Alves, Camilo Zurita-Salinas, Ecuadorian Bacteremia

**Affiliations:** Unidad de Investigaciones en Biomedicina. Zurita & Zurita Laboratorios, Quito, Pichincha, Ecuador; Unidad de Investigaciones en Biomedicina. Zurita & Zurita Laboratorios, Quito, Pichincha, Ecuador; Unidad de Investigaciones en Biomedicina. Zurita & Zurita Laboratorios, Quito, Pichincha, Ecuador; Mass General Brigham Salem Hospital, Salem, Massachusetts; Mass General Brigham Salem Hospital, Salem, Massachusetts; Unidad de Investigaciones en Biomedicina. Zurita & Zurita Laboratorios, Quito, Pichincha, Ecuador; Ecuadorian Bacteremia Working Group, Quito, Pichincha, Ecuador

## Abstract

**Background:**

Over the last years, several studies on bloodstream infections (BSI) have ranked *P. aeruginosa* in the top 5 list of Gram-negative causal agents. However, there is very limited data regarding the incidence of *Pseudomonas* bacteremia in Latin America. The aim of this study was to establish the incidence of bacteremia caused by *Pseudomonas* species

**Methods:**

During the period from November 2021 to May 2022 in 9 hospitals in Ecuador, blood culture samples were taken according to the clinical requirements of each patient. The bacteria identification was carried out using automated system (Vitek2 and Phoenix1000) and was confirmed by proteomic (MALDITOF) and molecular sequencing. Antimicrobial susceptibility was performed according to CLSI 2021.

**Results:**

A total of 297 gram-negative bacilli BSI were identified across the 9 hospitals where the study took place. Blood cultures positive for *Pseudomonas spp*. represented just 0.043% (13/297) of all bacteremia (Table 1). Four of the thirteen *Pseudomonas* presented a MDR profile. Resistance to carbapenems was higher than that observed in antipseudomonal cephalosporins (Table 2). *P*. *aeruginosa* species was the most prevalent variant (9/13) 69.2%, however other species such as *P. stutzeri, P. libanensis, P. mosselii, and P. otitidis* were also identified. It is important to emphasize that automated system failed to recognize these species. These four *Pseudomonas* species have not been reported before in Ecuadorian hospitals, most likely because in many hospitals MALDITOF and genomics are not routinely performed. The population demographic profile is shown in Table 3. Among the 13 patients with confirmed *Pseudomonas spp*. BSI, presumed bacteremia source varied among the samples.

**Figure:**
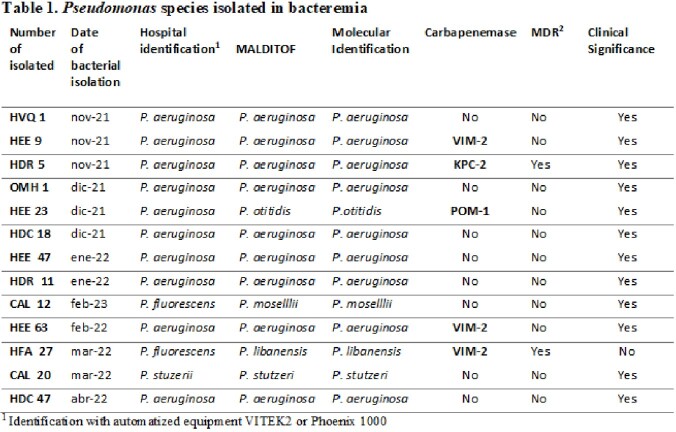

**Figure:**
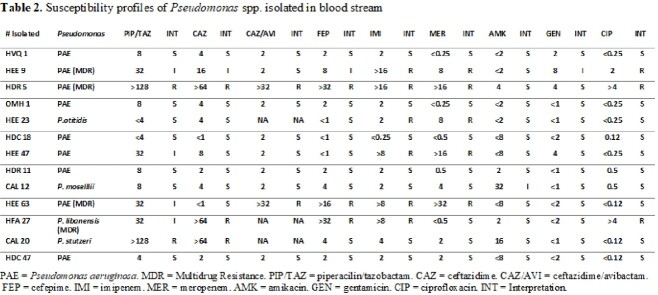

**Figure:**
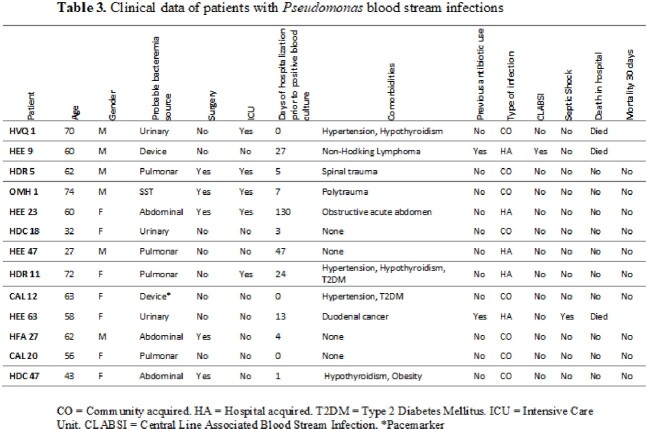

**Conclusion:**

This study provided relevant information on BSI caused by *Pseudomonas* in Ecuador.There are only few population-based studies evaluating bacteremia epidemiology in Latin America. SENTRY in 1997 estimated that *P. aeruginosa* contributed to 10.6% of gram-negative nosocomial and community-acquired bloodstream infections. Lower incidence levels, as seen in this study (0.043%), could help better understand population trends and help guide treatment.

**Disclosures:**

**Jeannete Zurita, n/a**, Pfizer: Grant/Research Support

